# Supplementary material for: Modulation of the Gut Microbiota by the Plantaricin-Producing Lactiplantibacillus plantarum D13, Analysed in the DSS-Induced Colitis Mouse Model
Source: Int J Mol Sci. 2023 Oct 18;24(20):15322. doi: 10.3390/ijms242015322 (PMC10607255; doi:10.3390/ijms242015322)
Supplement: Supplementary file 1 [file ijms-24-15322-s001.zip › Supplementary files/Supplementary Table 1.pdf]

Table S1. Antimicrobial activity of *L. plantarum* strains against *S. aureus* ATCC® 25923™ and *L. monocytogenes* ATCC® 19111™ evaluated by agar spot test assays expressed as effective inhibition ratio (EIR). Antimicrobial activity was interpreted as strong when EIR > 1.5, medium when 0.5 < EIR < 1.5, or weak when EIR < 0.5.

**Strain**	***S. aureus* ATCC® 25923™**	***L. monocytogenes* ATCC® 19111™**
	**EIR**	
L. plantarum D4	1.60 (±0.14)	0.89 (±0.10)
L. plantarum D5	1.89 (±0.10)	1.57 (±0.12)
L. plantarum D7	1.75 (±0.05)	1.26 (±0.12)
L. plantarum D13	2.21 (±0.09)	1.78 (±0.10)
L. plantarum M4	2.12 (±0.19)	1.47 (±0.15)
L. plantarum M5	2.33 (±0.29)	1.55 (±0.14)
L. plantarum MA2	2.40 (±0.17)	0.83 (±0.11)
L. plantarum MA3	2.29 (±0.17)	0.82 (±0.16)
L. plantarum SF15C	2.39 (±0.16)	1.99 (±0.11)
L. plantarum ZG1C	2.45 (±0.18)	1.83 (±0.11)
L. plantarum M92C	2.50 (±0.10)	2.18 (±0.11)
L. plantarum L4	1.86 (±0.15)	1.86 (±0.15)
