# Supplementary material for: Modulation of the Gut Microbiota by the Plantaricin-Producing Lactiplantibacillus plantarum D13, Analysed in the DSS-Induced Colitis Mouse Model
Source: Int J Mol Sci. 2023 Oct 18;24(20):15322. doi: 10.3390/ijms242015322 (PMC10607255; doi:10.3390/ijms242015322)
Supplement: Supplementary file 1 [file ijms-24-15322-s001.zip › Supplementary files/Supplementary Table 2.pdf]

Table S2. Antimicrobial activity of differentially treated cell free supernatants (CFSs) of *L. plantarum* strains against *S. aureus* ATCC® 25923™ and *L. monocytogenes* ATCC® 19111™, determined by the well diffusion method. [ab]Different letter means statistically significant difference ($p < 0.05$) within the same column among the treatments compared to the control of CFSs of the same *Lactobacillus strain*. Statistical analysis was carried out using One Way ANOVA and the results are reported as mean value ± SD of three independent experiments.

Strain	CFS	Diameter of inhibition zone (mm)	
		***S. aureus* ATCC® 25923™**	***L. monocytogenes* ATCC® 19111™**
***L. plantarum* D13**	untreated - control	12.67 ± 0.58[a]	12.67 ± 0.58[a]
	proteinase K	10.67 ± 0.58[b]	10.67 ± 0.58[b]
	100 °C/30 min	10.67 ± 0.58[b]	11.00 ± 0.00[b]
	pepsin	9.50 ± 0.00[b]	10.50 ± 0.71[b]
	pancreatin	10.75 ± 0.35[b]	10.00 ± 0.00[b]
***L. plantarum* M5**	untreated - control	11.00 ± 0.00[a]	11.67 ± 0.58[a]
	proteinase K	10.33 ± 0.58[a]	11.00 ± 1.00[a]
	100 °C/30 min	11.00 ± 0.00[a]	10.67 ± 0.58[a]
	pepsin	11.00 ± 0.00[a]	11.50 ± 0.71[a]
	pancreatin	10.75 ± 0.35[a]	10.25 ± 0.35[b]
***L. plantarum* SF15C**	untreated - control	12.00 ± 0.00[a]	12.67 ± 0.58[a]
	proteinase K	10.67 ± 0.58[b]	10.67 ± 0.58[b]
	100 °C/30 min	10.67 ± 0.58[b]	11.33 ± 0.58[b]
	pepsin	9.50 ± 0.71[b]	10.50 ± 0.00[b]
	pancreatin	9.50 ± 0.71[b]	10.00 ± 0.00[b]
***L. plantarum* ZG1C**	untreated - control	10.67 ± 0.58[a]	11.67 ± 0.58[a]
	proteinase K	10.33 ± 0.58[a]	11.00 ± 0.00[a]
	100 °C/30 min	10.33 ± 0.58[a]	11.33 ± 0.58[a]
	pepsin	10.00 ± 0.00[a]	9.50 ± 0.00[b]
	pancreatin	10.50 ± 0.00[a]	10.25 ± 0.35[b]
***L. plantarum* M92C**	untreated - control	11.33 ± 0.58[a]	11.67 ± 0.58[a]
	proteinase K	10.00 ± 0.00[b]	10.33 ± 0.58[b]
	100 °C/30 min	11.00 ± 0.00[a]	11.33 ± 0.58[a]
	pepsin	10.00 ± 0.00[b]	10.25 ± 0.35[b]
	pancreatin	10.00 ± 0.00[b]	10.00 ± 0.00[b]
***L. plantarum* L4**	untreated - control	11.00 ± 1.00[a]	10.33 ± 0.58[a]
	proteinase K	10.33 ± 0.58[a]	10.00 ± 0.00[a]
	100 °C/30 min	10.67 ± 0.58[a]	10.00 ± 0.00[a]
	pepsin	10.00 ± 0.00[a]	9.75 ± 0.35[a]
	pancreatin	9.70 ± 0.35[a]	9.50 ± 0.71[a]
