# Supplementary material for: Modulation of the Gut Microbiota by the Plantaricin-Producing Lactiplantibacillus plantarum D13, Analysed in the DSS-Induced Colitis Mouse Model
Source: Int J Mol Sci. 2023 Oct 18;24(20):15322. doi: 10.3390/ijms242015322 (PMC10607255; doi:10.3390/ijms242015322)
Supplement: Supplementary file 1 [file ijms-24-15322-s001.zip › Supplementary files/Supplementary Table 3.pdf]

Table S3. Identification of putative plantaricins from sequenced PCR products of *L. plantarum* D13 strain using the NCBI database.

used primer	Blast score	E value	Perc. Identity	Accession No
plnA (forward)	*L. plantarum pln* A, B, C and D genes	0.0	99.76	X75323.1
plnA (reverse)	*Lactobacillus plantarum* subsp. *plantarum* strain YM-4-3 *pln* locus, partial sequence	0.0	99.27	JQ933106.1
plnEF (forward)	*Lactobacillus plantarum* subsp. *plantarum* *pln*I, *pln*E, *pln*F genes for plantaricin I, plantaricin E, plantaricin F, partial and complete cds, strain: E1	0.0	98.21	LC191869.1
plnEF (reverse)	*Lactobacillus plantarum* subsp. *plantarum* *pln*I, *pln*E, *pln*F genes for plantaricin I, plantaricin E, plantaricin F, partial and complete cds, strain: E1	0.0	98.21	LC191869.1
plnJ (forward)	*Lactobacillus plantarum pln* locus, strain C11	0.00	99.08	X94434.2
plnJ (reverse)	*Lactobacillus plantarum pln* locus, strain C11	0.00	99.30	X94434.2
