# Supplementary material for: Modulation of the Gut Microbiota by the Plantaricin-Producing Lactiplantibacillus plantarum D13, Analysed in the DSS-Induced Colitis Mouse Model
Source: Int J Mol Sci. 2023 Oct 18;24(20):15322. doi: 10.3390/ijms242015322 (PMC10607255; doi:10.3390/ijms242015322)
Supplement: Supplementary file 1 [file ijms-24-15322-s001.zip › Supplementary files/Supplementary Table 4.pdf]

Table S4. *In silico* identification of plantaricin producing genes and their putative biochemical functions, i.e. biosynthesis, structure, immunity and transport of plantaricins from the cell, obtained by RAST server in *L. plantarum* D13 strain.

Contig	Start (bp)	Stop (bp)	Function
			**Bioynthesis**
7	116925	116182	Three-component quorum-sensing regularory system, response regulator
7	117787	117044	Three-component quorum-sensing regularory system, response regulator
7	118582	117788	Three-component quorum-sensing regularory system, sensor histidin kinase
7	119453	119307	Three-component quorum-sensing regularory system, inducing peptide for bacteriocin biosynthesis
7	107366	107073	plantaricin biosynthesis protein PlnY (putative)
12	2536	1337	plantaricin biosynthesis protein PlnO
			**Structure**
7	114652	114822	bacteriocin precursor peptide PlnE (putative)
7	114847	115005	bacteriocin precursor peptide PlnF (putative)
12	2821	2654	bacteriocin precursor peptide PlnN (putative)
12	4011	4178	bacteriocin precursor peptide PlnJ (putative)
12	4209	4382	bacteriocin precursor peptide PlnK (putative)
7	110753	110063	integral membrane protein PlnT, membrane-bound protease CAAX family
7	108467	107781	integral membrane protein PlnW, membrane-bound protease CAAX family
7	109241	108561	integral membrane protein plnV, membrane-bound protease CAAX family
7	109996	109328	integral membrane protein PlnU, membrane-bound protease CAAX family
			**Transport**
7	112219	110843	Bacteriocin ABC-transporter, putative component
7	114385	112223	Bacteriocin ABC-transporter, ATP-binding and permease component
			**Immunity**
12	3149	2949	immunity protein PlnM
7	115104	115877	immunity protein PlnI, membrane-bound protease CAAX family
12	1306	560	immunity protein PlnP, membrane-bound protease CAAX family
14	304646	303885	immunity protein PlnP, membrane-bound protease CAAX family
12	4379	5047	immunity protein PlnL
