# Supplementary figures and images for: Modulation of the Gut Microbiota by the Plantaricin-Producing Lactiplantibacillus plantarum D13, Analysed in the DSS-Induced Colitis Mouse Model
Source: Int J Mol Sci. 2023 Oct 18;24(20):15322. doi: 10.3390/ijms242015322 (PMC10607255; doi:10.3390/ijms242015322)

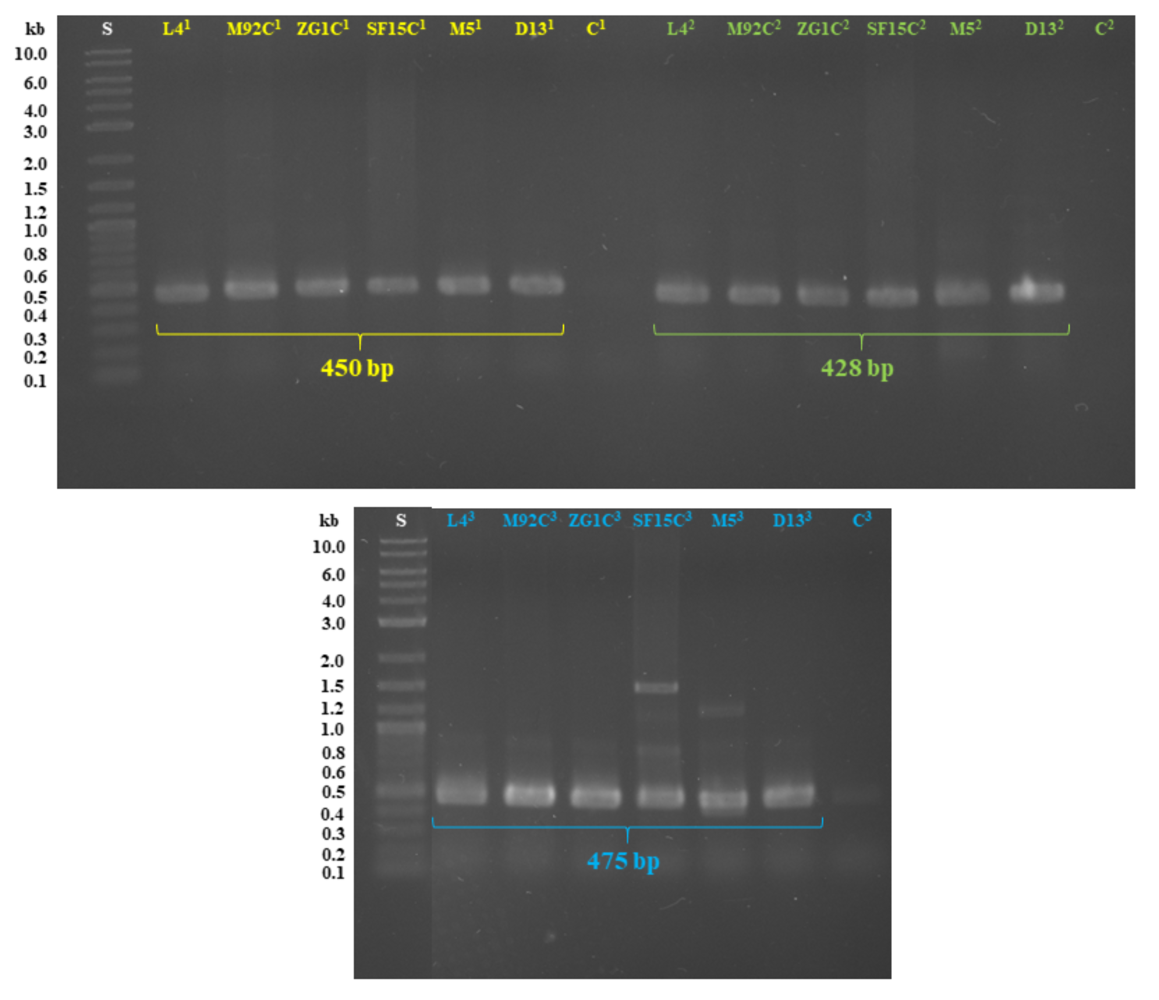

Supplement: Supplementary file 1 [file ijms-24-15322-s001.zip › Supplementary files/Supplementary Figure 1.tiff]
